# Complete Genome Sequence of the Multidrug-Resistant Neonatal Meningitis Escherichia coli Serotype O75:H5:K1 Strain mcjchv-1 (NMEC-O75)

**DOI:** 10.1128/MRA.01043-18

**Published:** 2018-09-13

**Authors:** Daniel W. Nielsen, Nicole Ricker, Nicolle L. Barbieri, James L. Wynn, Oscar G. Gómez-Duarte, Junaid Iqbal, Lisa K. Nolan, Heather K. Allen, Catherine M. Logue

**Affiliations:** a Department of Veterinary Microbiology and Preventive Medicine, Iowa State University College of Veterinary Medicine, Ames, Iowa, USA; b Food Safety and Enteric Pathogens Research Unit, National Animal Disease Center, ARS-USDA, Ames, Iowa, USA; c Department of Population Health, University of Georgia College of Veterinary Medicine, Athens, Georgia, USA; d Department of Pediatrics and Pathology, Immunology, and Laboratory Medicine, University of Florida, Gainesville, Florida, USA; e Division of Pediatric Infectious Diseases, Department of Pediatrics, University at Buffalo, The State University of New York, Buffalo, New York, USA; f Department of Infectious Diseases, University of Georgia College of Veterinary Medicine, Athens, Georgia, USA; University of Delaware

## Abstract

Neonatal meningitis Escherichia coli (NMEC) is the second leading cause of neonatal bacterial meningitis worldwide. We report the genome sequence of the multidrug-resistant NMEC serotype O75:H5:K1 strain mcjchv-1, which resulted in an infant’s death. The O75 serogroup is rare among NMEC isolates; therefore, this strain is considered an emergent pathogen.

## Abstract (precis)

Neonatal meningitis Escherichia coli (NMEC) is the second leading cause of neonatal bacterial meningitis worldwide. We report the genome sequence of the multidrug-resistant NMEC serotype O75:H5:K1 strain mcjchv-1, which resulted in an infant’s death.

## ANNOUNCEMENT

Although neonatal meningitis Escherichia coli (NMEC) is the second leading cause of neonatal bacterial meningitis (NBM), after group B streptococci, NMEC is responsible for the greatest mortality in NBM cases (1–3). The NMEC serotype O75:H5:K1 strain mcjchv-1 (NMEC-O75) described here was isolated from a 30-day-old newborn with meningitis. The patient subsequently died of infection complications after 16 days of hospitalization despite appropriate antibiotic management (4). Although NMEC strains RS218 (5), CE10 (6), IHE3034 (7), NMEC O18 (8), and S88 (9) have been sequenced and are publicly available in the NCBI GenBank database, these isolates all belong to serogroups O7 and O18, and a depth of clinical information about the strains is not readily available. Here, we present the genome sequence of NMEC-O75, a clinical isolate with a previously published clinical history (4).

NMEC-O75 was grown on MacConkey agar and subsequently in Luria-Bertani broth at 37˚C. Genomic DNA (gDNA) was extracted using the DNeasy blood and tissue Genomic-tip kit (Qiagen, Hilden, Germany) for Pacific Biosciences (PacBio, Menlo Park, CA) sequencing and the ChargeSwitch gDNA mini bacteria kit (Life Technologies, Carlsbad, CA) for Illumina sequencing. DNA yields were quantified using a Qubit fluorimeter double-stranded DNA (dsDNA) HS kit (Life Technologies). The Nextera Flex kit (Illumina, San Diego, CA) was used to prepare the genomic library for MiSeq sequencing, and the SMRTbell kit (Pacific Biosciences) was used to prepare the genomic library for PacBio sequencing with BluePippin (Sage Science, Beverly, MA) size selection to target a library size of 10 kb.

Genomic sequencing was performed on the PacBio (Menlo Park, CA) RS II and MiSeq (Illumina, San Diego, CA) instruments. Three single-molecule real-time (SMRT) cells were used for PacBio sequencing before the long reads were assembled with Canu version 1.5 (10). Next, the PacBio assembly was circularized in Geneious version 10.2 (11). Illumina reads that had been trimmed with Trimmomatic version 0.36 were used to further correct and polish the PacBio assembly with Pilon version 1.22 (Broad Institute) (12, 13). The trimmed Illumina reads were assembled with SPAdes (14), and potential contigs with plasmids were identified via the PlasmidFinder database (15) in ABRicate from polished PacBio and SPAdes assemblies (https://github.com/tseemann/abricate). tRNA-carrying and protein-encoding genes were assessed via Prokka version 1.13 (16). A complete workflow can be found at http://github.com/nielsend/genomeassembly.

The NMEC-O75 genome consists of a single chromosome, one large plasmid, and four small plasmids. The chromosome consists of 4,939,457 bp, with 50.6% GC content. It encodes 91 tRNAs and contains 4,593 coding sequences. The large plasmid, pNMEC-O75A, is a hybrid IncFIA/IncFIB plasmid. It consists of 88,420 bp and 50.6% GC content and contains 97 coding sequences. The four small plasmids, pNMEC-O75B, pNMEC-O75C, pNMEC-O75D, and pNMEC-O75E, range from 1,983 to 6,465 bp and have 42.6 to 56.1% GC content. Genomic comparisons of NMEC-O75 with other NMEC genomes are ongoing.

### Data availability.

The chromosome and plasmids have been deposited in GenBank under the accession numbers CP030111, CP030112, CP030113, CP030114, CP030115, and CP030116.
